# Impact of global warming on Raynaud’s phenomenon: a modelling study

**DOI:** 10.12688/f1000research.24939.1

**Published:** 2020-07-30

**Authors:** Charles Khouri, Matthieu Roustit, Jean-Luc Cracowski

**Affiliations:** 1 Clinical pharmacology, Grenoble Alpes University Hospital, Grenoble, France; 2 Centre Regional de pharmacovigilance, Grenoble Alpes University Hospital, Grenoble, France; 3 HP2, U1042, University Grenoble Alpes, Grenoble, France

**Keywords:** Raynaud's phenomenon, global warming

## Abstract

**Background:** Raynaud’s phenomenon is induced by excessive vasoconstriction of the peripheral microcirculation in response to environmental factors, essentially cold, but also stress or emotions. The objective of the present study is to evaluate the impact of global warming on the worldwide prevalence and severity of Raynaud’s phenomenon over the 21 ^st^ century.

**Method:** We first estimated the correlation between average temperature and prevalence and severity of Raynaud’s phenomenon. Then, we mapped the prevalence and the severity of Raynaud’s phenomenon worldwide at Christmas 1999 using historical data and, using climate projections from the Inter-Sectoral Impact Model Intercomparison Project, we predicted the prevalence and severity of Raynaud’s phenomenon at Christmas 2099 according to four greenhouse-gas emission scenarios.

**Results**: The prevalence of Raynaud’s phenomenon in the general population is expected to decrease by 0.5% per degree Celsius increase. Furthermore, patients are expected to suffer from one less attack per week for each increase of 2.5 degrees Celsius.

**Conclusions**: Our study shows that global warming may have a significant impact on the prevalence and the severity of Raynaud’s phenomenon over the 21 ^st^ century. However, as expected, this will greatly depend on the level of greenhouse-gas emissions.

Raynaud’s phenomenon is induced by excessive vasoconstriction of the peripheral microcirculation in response to environmental factors, essentially cold, but also stress or emotions ^1^. Primary, or idiopathic, Raynaud’s phenomenon is the most frequent form (80–90%), while in some cases Raynaud’s phenomenon can be secondary to various auto-immune diseases (such as systemic sclerosis or systemic lupus erythematous) or drugs ^1^. The prevalence of Raynaud‘s phenomenon is estimated to be approximatively 3 to 5% in the general population, with substantial variability according to climate and sex ^2^. Exposure to cold increase sympathetic adrenergic outflow inducing cutaneous vasoconstriction by constricting skin toes and fingers arteriovenous anastomoses ^3^. In individual with Raynaud’s phenomenon, the already-heightened sympathetic vasoconstriction is further amplified in intensity and will precipitate vasospasm of this vascular network ^2, 3^. Therefore sudden temperature change but also mean environmental temperature are determinant of Raynaud’s phenomenon burden and strong seasonal variation are described ^4– 6^. Most vasodilators currently used in Raynaud’s, such as nifedipine or sildenafil, only have limited efficacy, below the minimal clinically important difference ^7^. Moreover, most recent trials have produced negative results, due to high heterogeneity and a significant placebo effect ^8^.

We hypothesize that global warming should not leave Raynaud’s phenomenon as an unmet clinical need for too long. The objective of the present study is to evaluate the impact of global warming on the worldwide prevalence and severity of Raynaud’s phenomenon over the 21 ^st^ century.

## Method

We first estimated the correlation between average temperature and the prevalence of Raynaud’s phenomenon. The prevalence data were extracted from a systematic review of observational studies ( Table 1) ^9^. For each study we calculated the mean temperature during the winter preceding the publication of the study (from 1 ^st^ November to 31 March) using historical climate data from the database developed by the Inter-Sectoral Impact Model Intercomparison Project (ISIMIP). The results were then extrapolated to other countries using latitudes coordinates.

**Table 1.** Extracted data from the systematic review of Garner *et al.* ^9^. Data is reproduced under the terms of the Creative Commons Attribution Non Commercial (CC BY-NC 4.0).

Study	Year	Country	City	Latitude	Sample size	Mean age	Mean RP frequency
Brand	1997	USA	Boston	42.36	4182	51.8	7.20
Fraenkel	1999	USA	Boston	42.36	1525	53.9	7.80
Harada	1991	Japan	Ehime	33.84	3873	20–70	1.60
Ivorra	2001	Spain	Valencia	39.47	276	54.4	3.30
Maricq	1997	USA	South Carolina	33.84	2518	>18	2.10
Maricq	1997	France	Toulon	43.12	2187	>18	7.10
Maricq	1997	France	Nyons	44.36	2341	>18	6.00
Maricq	1997	France	Grenoble	45.19	2341	>18	9.25
Maricq	1997	France	Tarentaise	45.37	2296	>18	11.05
Onbasi	2005	Turkey	Van	38.50	768	29.2	5.90
Heslop	1983	UK	Southampton	50.91	450	20–59	12.70
Purdie	2009	New Zealand	Wellington	41.25	234	>18	11.50
Sahin	2003	Turkey	Van	38.50	251	28.9	3.98
Leppert	1987	Sweden	Vasteras	59.61	2705	18–59	11.00
Olsen	1978	Denmark	Copenhagen	55.68	67	21–50	15.51
Tzilalis	2011	Greece	Athens	37.98	3912	18–28	0.31
Cakir	2008	Turkey	Edirne	41.15	1414	27.2	3.60
Gallo	1994	Italy	Milan	45.46	1920	15–84	4.20
Voulgari	2000	Greece	Ioannina	39.77	500	33.7	5.20
Jones	2003	UK	Manchester	53.48	716	12–15	14.90

We further predicted the impact of global warming on the severity of Raynaud’s phenomenon, expressed as the average daily frequency of attacks, by using a model based on a Poisson regression including temperature (and other covariates), recently published by our team ^10^ (this model is available online from DRYAD). This model is derived from a series of n-of-1 trials containing more than 2000 days of exposition, with daily temperature measurements collected at the nearest weather station to the patient’s home.

Finally, we mapped the prevalence and the severity of Raynaud’s phenomenon worldwide at Christmas 1999 and, using climate projections from the ISIMIP, we predicted the prevalence and severity of Raynaud’s phenomenon at Christmas 2099, according to four greenhouse-gas emission scenarios (Representative Concentration Pathway (RCP) 2.6, RCP4.5, RCP6.0, and RCP8.5) described in the Fifth Assessment Report of the United Nations Intergovernmental Panel on Climate Change ^11^. The HadGEM2-ES model was used for the modelling scenario ^12^.

The RCPs represent the range of greenhouse-gas emission scenarios consistent with projections described in the literature; they include a mitigation scenario (RCP2.6), two intermediate scenarios (RCP4.5 and RCP6.0), and one scenario with high greenhouse-gas emissions (RCP8.5).

Data analysis were performed with R version 3.3.0 ^13^ and map visualization with Panoply version 4.10.4 software ^14^.

A patient, Mrs Laurence Schuller, member of the board of the French Scleroderma Patient Association, was invited to comment on the study design and to interpret the results.

## Results

We found a high correlation between average temperature and the prevalence and severity of Raynaud’s phenomenon (p<0.001). According to these data, no Raynaud’s phenomenon attack is expected to occur above an average temperature of 13°C, which is consistent with individual data collected in our series of N-of-1 trials ^10^. Consequently, the prevalence of Raynaud’s phenomenon in the general population is expected to decrease by 0.5% per degree Celsius increase. Furthermore, patients are expected to suffer from one less attack per week for each increase of 2.5 degrees Celsius.

**Figure 1.**
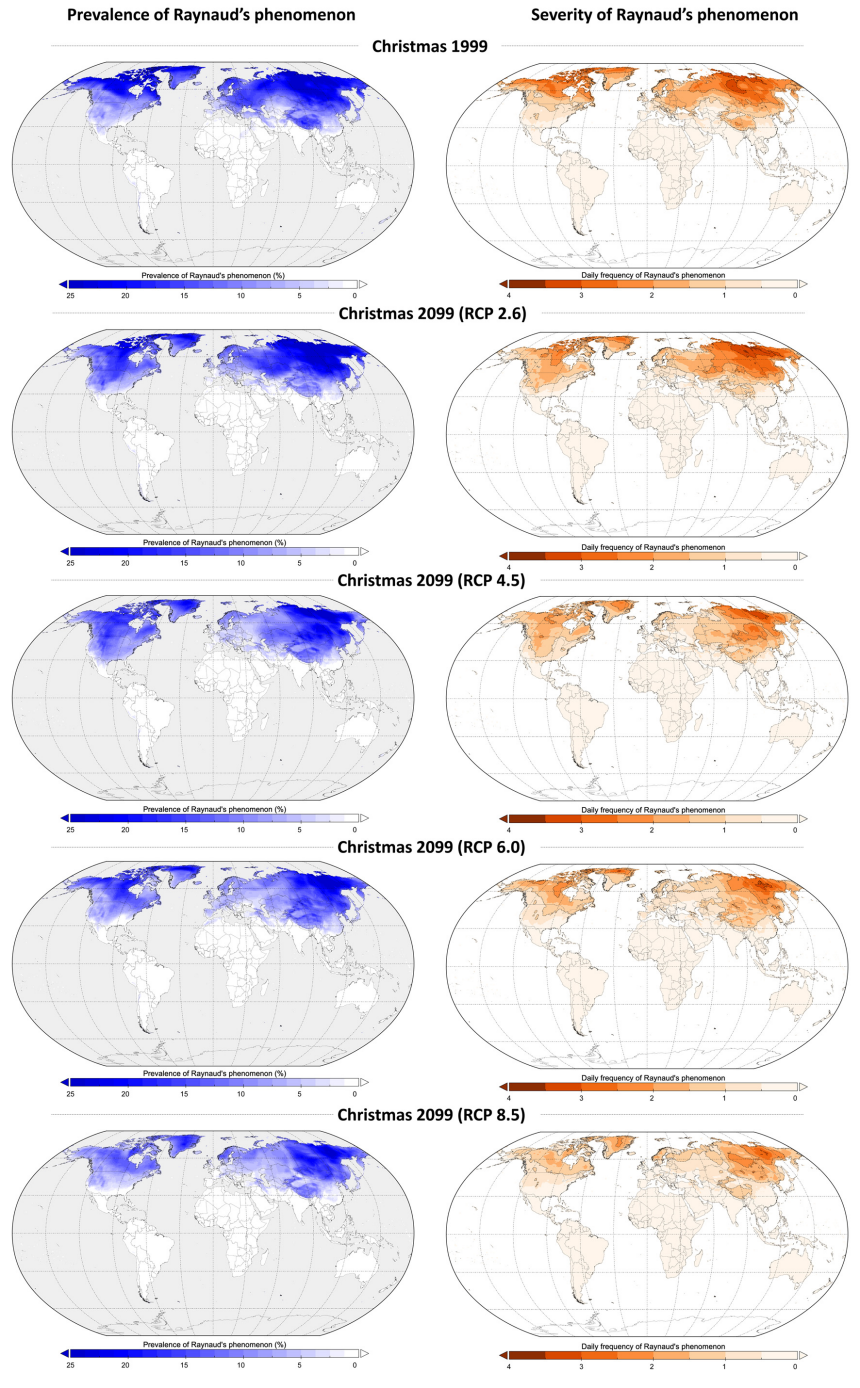
Prevalence and daily frequency of Raynaud’s phenomenon during Christmas 1999 and Christmas 2099 according to four greenhouse gas emission scenarios (Representative Concentration Pathway (RCP) 2.6, RCP4.5, RCP6.0, and RCP8.5).

 The worldwide prevalence and severity of Raynaud’s phenomenon at Christmas 1999 and the range of predictions based on four greenhouse-gas emission scenarios at Christmas 2099 are shown in Figure 1.

## Discussion

Our study shows that global warming may have a significant impact on the prevalence and the severity of Raynaud’s phenomenon over the 21 ^st^ century. However, as expected, this will greatly depend on the level of greenhouse-gas emissions. The most optimistic greenhouse gas scenario (RCP 2.6), which aims at keeping global warming below 2°C above pre-industrial temperatures, only has a limited impact on the global prevalence and severity of Raynaud’s phenomenon. On the other hand, scenarios without greenhouse-gas emission reductions (predictions ranging between RCP6.0 and RCP8.5) may largely improve the condition of patients suffering from Raynaud’s phenomenon. For example, people in western European countries could expect to be totally free of this painful and disabling condition in the event of the two higher gas-emission scenarios. Finally, patients in North America, Western Europe and Asia still suffering from Raynaud’s phenomenon are not expected to suffer more than one or two crises over the Christmas period in 2099.

A limitation to our model is that we did not consider the potential increase in the use of air-conditioning and the stress caused by global warming, which may enhance RP. Climate change is also likely to result in temperature anomalies, including rapid temperature fluctuations which are known to be triggering factors of Raynaud’s phenomenon attacks. Nevertheless, mean temperatures are correlated to RP prevalence. In this study we only used one modelling scenario, the HadGEM2-ES model, which is widely used for climate research ^15, 16^, therefore uncertainty of our projections has not been evaluated but exist undoubtedly. The findings should thus be interpreted as potential impacts of climate change on Raynaud’s phenomenon according to one hypothetical scenario and not as projections.

## Conclusion

In conclusion, this study shows that global warming is likely to have a significant impact on the prevalence and the severity of Raynaud’s phenomenon. Yet, whether the advantages of global warming will outweigh its drawbacks, even for Raynaud’s phenomenon patients, remains to be carefully scrutinized.

## Data availability

### Source data

The data from the N-of-1 trial PROFIL are freely available on datadryad.org

DRYAD: Data from: On-demand sildenafil as a treatment for Raynaud phenomenon: a series of n-of-1 trials. https://doi.org/10.5061/dryad.c670tq2 ^17^

The files required are:

- PROFIL_DATA (The dataset of the study in plain text format with variables names as header 2306 observations on 50 variables)
-model_1 (Final model)

Data are available under the terms of the Creative Commons Zero "No rights reserved" data waiver (CC0 1.0 Public domain dedication).

Historical and climate projections are available from the ISIMIP Earth System Grid Federation ( **ESGF**) server in searching the climate forcing “HadGEM2-ES” and the variable “tasAdjust”:

- tas_day_HadGEM2-ES_historical_r1i1p1_EWEMBI_landonly_19910101–20001231.nc (historical data)
- tas_day_HadGEM2-ES_rcp26_r1i1p1_EWEMBI_landonly_20910101–21001231 (climate projection for RCP 2.6)
- tas_day_HadGEM2-ES_rcp26_r1i1p1_EWEMBI_landonly_20910101–21001231 (climate projection for RCP 4.5)
- tas_day_HadGEM2-ES_rcp26_r1i1p1_EWEMBI_landonly_20910101–21001231 (climate projection for RCP 6.0)
- tas_day_HadGEM2-ES_rcp26_r1i1p1_EWEMBI_landonly_20910101–21001231 (climate projection for RCP 8.5)
