# Supplementary material for: Impact of global warming on Raynaud’s phenomenon: a modelling study
Source: F1000Res. 2020 Jul 30;9:829. [Version 1] doi: 10.12688/f1000research.24939.1 (PMC8220350; doi:10.12688/f1000research.24939.1)
Supplement: Peer review history

# Peer review: Reviewer response for version 1

**DOI:** 10.5256/f1000research.27514.r86792

**Published:** 2021-06-22

**Authors:** Rebecca S. Overbury

**Affiliations:** 1 Department of Internal Medicine, Division of Rheumatology, University of Utah, Salt Lake City, UT, USA

This is a research article that attempts to hypothetically model how rising temperatures, assumed due to increased levels of greenhouse gas emissions, would affect the prevalence and severity (as defined by frequency of "crises") of Raynaud's phenomenon. Overall, this is a truly fascinating, if disturbing, question to consider and one that is likely to be relevant for worldwide clinicians moving forward. The authors present previous research supporting a sound hypothesis from which to ask this question. I find this a novel and interesting research question. 

 I do not have expertise to speak to the methods of modelling in particular. However, the authors are transparent, if succinct, in explaining their methods and all necessary materials for reproducibility appear to be available. Someone well-versed in this may desire more detail.

 As a Rheumatologist, I recognize that the affect of this climate change phenomenon in patients with primary versus secondary Raynaud's is likely to differ; and I would be interested to hear more about this. This is not addressed here, but is arguably outside the scope of this methodology.

Is the work clearly and accurately presented and does it cite the current literature?

Yes

If applicable, is the statistical analysis and its interpretation appropriate?

Yes

Are all the source data underlying the results available to ensure full reproducibility?

Yes

Is the study design appropriate and is the work technically sound?

Yes

Are the conclusions drawn adequately supported by the results?

Yes

Are sufficient details of methods and analysis provided to allow replication by others?

Yes

Reviewer Expertise:

Rheumatology

I confirm that I have read this submission and believe that I have an appropriate level of expertise to confirm that it is of an acceptable scientific standard.

# Peer review: Reviewer response for version 1

**DOI:** 10.5256/f1000research.27514.r73786

**Published:** 2020-11-09

**Authors:** Rossella De Angelis

**Affiliations:** 1 Department of Clinical and Molecular Sciences, Rheumatology Unit, Carlo Urbani Hospital, Polytechnic University of Marche, Ancona, Italy

The predicted prevalence and the severity of Raynaud's phenomenon at Christmas 2099, according to four greenhouse gas emission scenarios as described in the 5th Assessment Report of United Nations.

 So:

1. If possible, a slightly detailed explanation of reference item 11 would be particularly welcome, also to make the data more understandable to doctors.
2. A rather more refined description of the statistical model HadGe2_ES (not only with a simple bibliographic citation), in order to make the reading of the work more fluent. On the other hand, a doctor who is interested in the study should go into the bibliographic item no. 12, which is not easily understandable at a first reading.

The work is, however, very interesting and describes a likely scenario. Those clinicians who deal with Raynaud's phenomenon should take this hypothesis into account. In any case, a welcome reading.

Is the work clearly and accurately presented and does it cite the current literature?

Yes

If applicable, is the statistical analysis and its interpretation appropriate?

Yes

Are all the source data underlying the results available to ensure full reproducibility?

Partly

Is the study design appropriate and is the work technically sound?

Yes

Are the conclusions drawn adequately supported by the results?

Yes

Are sufficient details of methods and analysis provided to allow replication by others?

Partly

Reviewer Expertise:

Rheumatology

I confirm that I have read this submission and believe that I have an appropriate level of expertise to confirm that it is of an acceptable scientific standard.
